# Editorial: Covid-19 therapies in patients with hematologic malignancies

**DOI:** 10.3389/fonc.2024.1382268

**Published:** 2024-02-19

**Authors:** Andrea Visentin, Massimo Gentile

**Affiliations:** ^1^ Hematology Unit, Department of Medicine, University of Padova, Padova, Italy; ^2^ Hematology Unit, Azienda Ospedaliera (AO) Cosenza, Cosenza, Italy; ^3^ Department of Pharmacy, Health and Nutritional Science, University of Calabria, Rende, Italy

**Keywords:** hematologic malignancies, COVID, tixagevimab/cilgavimab, vaccine, rituximab

The pandemic caused by the coronavirus SARS-CoV-2 disease (CODIV-19), has significantly impacted global health since its emergence in late 2019. While the virus primarily targets the respiratory system, its effects extend beyond the lungs, affecting various organs and systems in the body finally causing post-COVID condition, also known as long-COVID (1). Individuals with underlying health conditions, including hematological malignancies, faced unique challenges when confronting COVID-19. Patients with hematological malignancies, in particular with chronic lymphocytic leukemia and multiple myeloma, often have compromised immune systems due to the nature of the diseases themselves or the treatments they undergo, such as chemotherapy, targeted therapies or stem cell transplantation.

One of the key concerns for patients with hematological malignancies during the COVID-19 pandemic is the increased vulnerability to severe illness. The weakened immune response can make it difficult for these patients to fight the infection, including the respiratory complications associated with COVID-19, resulting in high rate of hospitalization, intensive care unit admission and death (1–3). Several studies also suggest that COVID-19 can exacerbate the complications of hematological malignancies, leading to disruptions in treatment schedules and heightened concerns about disease management. Furthermore, the long-term effects of the virus on the immune system of individuals with these malignancies remain a subject of ongoing research.

In this regard, healthcare providers had to adopt stringent measures to protect these individuals, emphasizing preventive strategies and closely monitoring their health. As a result, vaccination campaigns targeting this vulnerable population have been crucial in reducing the severity of COVID-19 infections and preventing complications (1, 2, Palumbo et al, Mohamed et al, 4, Autore et al). Ongoing studies aim to elucidate the specific risks and outcomes associated with COVID-19 in this patient population, as well as the effectiveness of active and passive therapies in conferring protection (Angotzi et al, Hijano et al, 5). The integration of data from diverse sources, including clinical trials and real-world evidence, is essential for informing evidence-based guidelines that can guide clinicians in managing hematological malignancies during the ongoing pandemic.

In this Research Topic we aim to better understand the intersection between COVID-19 and hematological malignancies in particular the use of SARS-CoV-2 protein inhibitors, monoclonal antibodies, vaccinations.

Mohamed et al. reported the final results of the CERVAX study. Only 33% of the patients seroconverted after the first dose of vaccine, which increased to 56% after the second dose. Non-serological responders (18%) were on active therapy with lenalidomide, ibrutinib or venetoclax. SARS-CoV2-reactive T-cell analysis by interferon gamma release assays also showed a response in 55% of the patients.

Mancuso et al. focused on 103 patients with multiple myeloma showing that despite a high seropositivity rate (88% after the second dose and 99% after the third dose of vaccine) only 36% of the patients had a T-cell response. Of interest, patients with a complete hematological response and lenalidomide maintenance showed an enhanced response to vaccination, as compared to patients receiving proteasome inhibitors/anti-CD38 monoclonal antibodies

Autore et al. did a multicenter retrospective observational study on 82 patients who underwent autologous stem cell transplantation, 58 and 39 were vaccinated after and before transplant, respectively. In patients with non-Hodgkin lymphoma previous treatment with rituximab predicted a negative serology. Instead among patients with a positive titer of anti-SARS-CoV2 before transplant became negative after transplantation. Suggesting that autologous stem cell transplantation did not affect the response to the vaccination.

Accordingly, Palumbo et al. found that patients with myelofibrosis and polycythemia vera receiving ruxolitinib had an impaired antibody response after 2 doses vaccines, as 32.5% of patients did not develop any response. However, after the third booster dose 80% of these patients produced antibodies above the threshold positivity. However, the quantity of produced antibodies was well below that reached than those reported for healthy individuals.

Two studies assessed the efficacy of pre-exposure prophylaxis with tixagevimab-cilgavimab. Angotzi et al. analyzed 103 adult patients with hematological malignancies, while Hijano et al. 27 children. In the former study, only patients with a negative anti-SARS-CoV2 antibody titer after vaccination received tixagevimab-cilgavimab (34%). The 3-month cumulative incidence of infection was 20% versus 12% in the tixagevimab-cilgavimab vs observation only groups (p=0.34), respectively. Twenty-seven percent of the pediatric patients developed SARS-CoV-2 infection within 180 days of receiving tixagevimab-cilgavimab. In both studies, only one patient required hospitalization, but no serious events were reported.

Vita et al. described a case of a 51 years old Italian woman with acute lymphoblastic leukemia who underwent to allogeneic hematopoietic stem cell transplantation during SARS-COV-2 infection, that was treated with dual anti SARS-COV-2 antiviral plus monoclonal antibody.

In conclusion, the COVID-19 pandemic has posed significant challenges for individuals with hematological malignancies, emphasizing the need for tailored approaches to care and management. The delicate balance between treating underlying diseases and mitigating the risks of COVID-19 requires ongoing research, collaboration among healthcare professionals, and a commitment to adapting strategies as new information emerges. The prioritization of the well-being of individuals with hematological malignancies remains a critical aspect of the broader public health response.

## Author contributions

AV: Writing – original draft, Writing – review & editing. MG: Writing – original draft, Writing – review & editing.
